# Green conversion of graphene oxide to graphene nanosheets and its biosafety study

**DOI:** 10.1371/journal.pone.0171607

**Published:** 2017-02-03

**Authors:** Adhiraj Dasgupta, Joy Sarkar, Manosij Ghosh, Amartya Bhattacharya, Anita Mukherjee, Dipankar Chattopadhyay, Krishnendu Acharya

**Affiliations:** 1 Molecular and Applied Mycology and Plant Pathology Laboratory, Department of Botany, University of Calcutta, Kolkata, West Bengal, India; 2 Department of Botany, Dinabandhu Andrews College, Garia, Kolkata, West Bengal, India; 3 Cell Biology & Genetic Toxicology Laboratory, Centre of Advanced Study in Cell & Chromosome Research, Department of Botany, University of Calcutta, Kolkata, West Bengal, India; 4 Department of Polymer Science & Technology, University College of Science & Technology, University of Calcutta, Kolkata, West Bengal, India; 5 Center for Research in Nanoscience & Nanotechnology, Technology Campus, University of Calcutta, Kolkata, West Bengal, India; Institute of Materials Science, GERMANY

## Abstract

Chemical reduction of graphene oxide (GO) to graphene employs the use of toxic and environmentally harmful reducing agents, hindering mass production of graphene which is of tremendous technological importance. In this study we report a green approach to the synthesis of graphene, bio-reduced by crude polysaccharide. The polysaccharide reduces exfoliated GO to graphene at room temperature in an aqueous medium. Transmission electron microscopy image provides clear evidence for the formation of few layer graphene. Characterization of the resulting polysaccharide reduced GO by Raman spectroscopy, Fourier transform infrared spectroscopy and Energy dispersive X-ray analysis confirms reduction of GO to graphene. We also investigated the degree of biosafety of the reduced GO and found it to be safe under 100 μg/ml.

## Introduction

Due to the electronic, mechanical, thermal and optical uniqueness, two-dimensional graphene nanosheets (GNS) have significantly transformed areas of nanoscience [1,2]. Even though recent research has produced some unique and versatile 2-D nanostructures [3], GNS still holds great promise for potential applications in nanoelectronics [4], sensors [5,6], nanocomposites [7] and other technological fields. Harnessing these characteristics, which renders GNS its potential, necessitates a large-scale production of the nanosheets. Micromechanical cleavage [1], epitaxial growth [8], solution-based chemical reduction [9] and a few other methods are mainly applied for production of GNS. Usually, chemical reduction of GO was carried out using hydrazine and its derivatives [10,11], but its high toxicity and instability makes the procedure potentially hazardous and asks for great care. GNS has a tendency of π – π stacking, making the bulk synthesis of it a key challenge. This can be overcome by the attachment of other molecules or polymers on to the nanosheets. Recently, scientists have reported synthesis of GNS under much milder conditions using molecules like ascorbic acid [12], reducing sugars like glucose and fructose [13], which are ecofriendly and very effective in reducing GO to GNS.

Carbon based nanoparticles, mainly graphite nanoformulations have seized a lot of fancy recently. Ground breaking progress is being made with these formulations and the potentials are infinite. Lately, some researchers have been able to synthesize an ultra light weight Carbon Microtube, Aerographite [14], with excellent mechanical performance. These microtubes were utilized to produce various 3D elastic hybrid networks [15] and ZnO hybrid nanomaterials which have unique and promising optoelectronic properties[16].

Carbon based nanomaterials including graphene and its derivatives have significant importance in biomedical applications. In the recent times, few researchers are concentrating on exploiting graphene based nanohybrids for electrochemical biosensing. Parlak et al. [17] have been able to construct a graphene-enzyme bioelectrode capable of biosensing. Further a graphene-based zipper-like interface has been reported [18] as an efficient bioelectrocatalyte. Although significant research has been conducted for carbon nanotubes, results on biosafety of graphene and its derivatives are relatively less. Among those reported, *in vitro* studies on BEAS-2B cells [19] and PC12 cells [20] demonstrated toxic effects of graphene oxide leading to apoptosis. Graphene oxide produced by Hummers method induced cell cycle alternation and apoptosis in Saos-2, MC3T3-E1 and RAW-264.7 cells [21]. However studies on HepG2 cells [22,23] revealed only moderate levels of toxicity. Interestingly, in another study graphene oxide nanoparticle coated with polyethylene glycol, did not induce toxic response to several cell lines (RAJI, HCT-116, OVCAR-3, U87MG, MCF-7) up to a concentration of 100 μg/ml [24–27]. A recent review by Seabra et al. [28], has elegantly described the toxicological impact of graphene and graphene oxide nanoparticles and highlighted the lack of homogeneity and consensus in findings. The small size of nanoparticles facilitates their uptake into cells as well as transcytosis across epithelial cells into blood and lymph circulation [29]. Only a limited number of studies have been conducted on the biocompatibility of graphene based nanomaterials on blood cells and the results are often contradictory [30–33]. Hence, prior to the use of novel graphene based nanomaterials, its biological compatibility needs to be investigated.

Mushrooms are presently rising as an elite source of biologically active molecules with promising use in the medical and food industries. They are especially rich in polysaccharides, many of which have been reported as potential immunomodulants [34–38]. In this study we report a simple green approach for reduction of GO in aqueous solution to GNS, which we refer to as polysaccharide-reduced graphene oxide (PR-GO) using water soluble polysaccharides from a wild edible mushroom *Pleurotus flabellatus* Sacc. Also, in course of evaluating the interaction of different commercial and biosynthesized nanoparticles with biomolecules at different trophic levels [39–44], we give an updated overview on the biosafety of the newly synthesized crude polysaccharide coated GNS, using cyto-genotoxic endpoints in human peripheral blood mononuclear cells (PBMCs).

## Materials and methods

### Extraction of polysaccharide rich fraction

The basidiocarps of *P*. *flabellatus* were dried, powdered and extracted with ethanol at 25°C for 2 days to eliminate triterpenoids, steroids and other alcohol soluble compounds. It was then filtered and the residue was similarly re-extracted. The air dried residue was steeped in boiling distilled water for eight hours to extract the water soluble biomolecules and filtered. Polysaccharides were precipitated with ethanol and centrifuged. The pellet was re-dissolved in distilled water and centrifuged. The clear supernatant was lyophilized to dryness using a Scanvac lyophilizer (Labogene). The dry polysaccharide was collected and stored in a desiccator for further use [45]

### Physico-chemical characterization of crude polysaccharide

Total carbohydrate, protein and phenol were determined using phenol-sulphuric acid, Bradford and Folin-Ciocalteau methods respectively and quantified using glucose, BSA and gallic acid as respective standards [45]. Total glucan, α-glucan and β-glucan were quantified using mushroom and yeast β-glucan assay kit (Megazyme Int.). Helical structure of the polysaccharide was analyzed by Congo red-polysaccharide reaction according to the method described by Qiu et al [46]. Sugar composition of the crude polysaccharide was partially determined by HPTLC [47] and GC-MS [48]. TFA-hydrolyzed polysaccharide was separated by HPTLC with chloroform, *n*-butanol, methanol, water and acetic acid (4.5: 12.5: 1.5: 1.5) as solvent system and 0.1% orcinol in 5% H_2_SO_4_ as detection reagent. Monosaccharides were derivatized to alditol-acetates and subjected to GC-MS (Agilent technologies, USA, HP5MS 30 mm × 0.25 mm × 0.25 μm + 10 m Duraguard column) under defined conditions (80°C for 2 min, then 15°C /min to 200°C for 2 min, then 4°C /min to 240°C for 2 min, then 15°C /min to 280°C for 5 min, pressure: 9.4 psi, constant flow rate of 1 ml /min, carrier gas: He). Chromatograms of the samples were analyzed by comparing results with standard carbohydrates as well as using NIST database stored in the system.

### Preparation of graphene oxide and reduction of GO to PR-GO nanosheets

The graphite oxide was prepared from graphite powder by Hummers method [49]. The obtained GO was dispersed in deionized water (2 mg/ml) and exfoliated in an ultrasonicator for two successive 30 min periods. This exfoliated GO was concomitantly added to an aqueous solution (20 mg/ml) of crude water-soluble polysaccharide and stirred for 48 hours under room temperature. The solution was monitored at regular intervals. Both positive (crude polysaccharide solution of *Pleurotus flabellatus*) and negative control (Graphene oxide solution) were maintained under similar conditions. Then the solution was centrifuged and the pellet was used for further characterization.

### Characterization of GO and PR-GO

Particle size of the PR-GO nanosheets was measured by laser diffractometry using a nano size particle analyzer (Zen 1600 Malvern USA) in the range between 0.6 nm and 6.0 μm. Transmission Electron Microscopy was performed on a Tecnai G^2^ spirit Biotwin (FP 5018/40), operating at an accelerating voltage of 80 kV. XRD measurements of PR-GO was taken with a PW 3040/60 PANalytical X-ray diffractometer that operated at a voltage of 45 KV and current of 30 mA with Cu Kα radiation (λ 1.54443 Ǻ). The diffracted intensities were recorded from 35° to 90° 2θ angles. EDX analysis was carried out with a Hitachi S 3400N, Japan, to identify the elemental compositions of the particles. The polysaccharide (PS), GO and PR-GO, were all analysed by Fourier transform infrared (FTIR) spectroscopy (Shimadzu 8400S fourier transform infrared spectrophotometer). The system worked in a diffuse reflectance mode at a resolution of 4 cm^-1^ in KBr pellets. The scanning data were obtained from the average of 50 scans in the range of 4000 to 400 cm^-1^. Raman spectra was monitored using 1.96 eV (633 nm) line of a He Ne laser in HORIBA-JOBIN-YVON Lab RAM HR 800 instrument by placing the sample solution into a semi-micro stopper cuvette with an exposure time of 1 s.

### Biosafety evaluation of PR-GO nanosheets in human lymphocyte cells

Lymphocyte cells were isolated from fresh blood according to the method of Boyum [50], using Histopaque. The cells were washed with PBS and resuspended in RPMI-1640 media at a concentration of 10^6^ cells/ml. The cells were incubated in RPMI-1640 media containing different concentrations of PR-GO nanosheets (0, 50, 100 and 250 μg/ml), for 3 h at 37°C. Following treatment, the lymphocytes were processed for cytotoxicity and genotoxicity assays. The lymphocytes were also processed for FACS analysis for oxidative stress.

#### Evaluation of cytotoxicity of PR-GO nanosheets by MTT assay

The MTT assay allowed the quantitative determination of cell viability. The assay is based on the capability of viable cells to convert MTT to spectroscopically detectible formazan. The assay was performed according to a method previously published [39]. after treatment and incubation, cells were treated with 0.5 mg/ml solution of 3-(4, 5-dimethylthiazolyl-2)-2,5-diphenyltetrazolium bromide (MTT; 100 μl/well) at 37°C for 3 h. Optical density (OD) was read on iMark™ Microplate Absorbance Reader (BIO-RAD, USA) at 570 nm, with 630 nm as a reference wavelength. The interference of nanosheets in the assays was eliminated by maintaining sample blanks for all the concentrations tested. The OD values obtained from the sample blanks were deducted from the OD values obtained after the assays. The values obtained thereafter had been expressed as percentage compared to control. All experiments were performed at least in triplicate on three separate occasions. Data have been presented as mean ± SD. Triton X-100 (0.1%, 10 min) was used as positive control for the experiments.

#### Evaluation of cytotoxicity of PR-GO nanosheets by resazurin assay

The resazurin system measures the metabolic activity of living cells [51]. Resazurin is reduced to resorufin (highly fluorescent) in the medium by cell activity, and a direct correlation exists between the reduction of resazurin in the growth medium and the metabolic activity of living cells. Cells after incubation were washed twice in PBS. Resazurin assay was performed using Resazurin based *in vitro* toxicology assay kit (TOX-8) according to methods previously reported [39]. To eliminate variability, three replicates per concentration were maintained. Triton X-100 (0.1%, 10 min) was used as positive control for the experiments.

#### Evaluation of cytotoxicity of PR-GO nanosheets by neutral red uptake assay

Neutral red uptake assay was done to determine the accumulation of neutral red dye in the lysosomes of viable, uninjured cells [52]. Neutral red retention assay was performed according to the methods described previously [39]. Following incubation with PR-GO, the cells were washed carefully with PBS and incubated with neutral red solution (4 μg ml^−1^ in PBS) for 3 h. Following incubation cells were rinsed (thrice) with PBS to remove all dye not incorporated within cellular lysosomes. 200 μl of acidified ethanol (1% acetic acid, 50% ethanol, 49% D H_2_O) was added to each well and incubated in dark for 15 min, before being read at 540 nm in the plate reader (iMark™ Microplate Absorbance Reader (BIO-RAD, USA). All experiments were performed at least in triplicate on three separate occasions. Triton X-100 (0.1%, 10 min) was used as positive control for the experiments.

#### Evaluation of cytotoxicity of PR-GO nanosheets by flow cytometric estimation of propidium iodide (PI) uptake

Following treatment with PR-GO (0, 50, 100 and 250 μg/ml) for 3 h at 37°C, cells were washed twice with PBS and re-suspended in 500 μl PBS (2 × 10^6^ cells, 25 μM PI). A set of three replicates per concentration were maintained for the experiment. After a fifteen-minute incubation at room temperature in the dark, the PI fluorescence in cells was measured by flow cytometry (FACS Aria III flow cytometer, BD Biosciences, USA; excitation λ = 488 nm, emission λ = 530–540 nm) and 20,000 events were acquired for analysis. Triton X-100 (0.1%, 10 min) was used as positive control. Results were expressed as the mean fluorescence intensity. The interference of nanosheets in the assays was eliminated by maintaining sample blanks for all the concentrations tested.

#### Evaluation of genotoxicity of PR-GO by comet assay

Human lymphocytes incubated with PR-GO nanoparticles (0, 50, 100 and 250 μg/ml) for 3 h at 37°C were processed for DNA damage studies following the method of Singh et al. [53], with modifications [41]. Slides were prepared in triplicates per concentration. Slides were immersed in cold lysis solution (pH 10; 2.5 M NaCl, 100 mM Na_2_EDTA, 10 mM Trizma base, 1% Triton X–100, 10% DMSO) and kept at 4°C for 60 min. After lysis the DNA was allowed to unwind in the electrophoresis buffer (300 mM NaOH: 1mM Na_2_EDTA at pH 13.5) for 20 min at 4°C. This was followed by electrophoresis conducted at a constant voltage of 26 V and 300 mA at 4°C. Slides were neutralized in 0.4 M Tris (pH 7.5) for 5 min and finally rinsed in water. H_2_O_2_ (25 μM, 10 min, 4°C in dark) treated cells were used as positive control for the experiment. Each experiment was repeated twice.

The slides were stained with EtBr (20 μg/ml) and rinsed in water to wash off excess stain. Slides were scored using image analysis system (Kinetic imaging; Andor Technology, Nottingham, UK) attached to a fluorescence microscope (Leica, Wetzlar, Germany) equipped with appropriate filters (N2.1). The microscope was connected to a computer through a charge- coupled device (CCD) camera to transport images to software (Komet 5.5) for analysis. The final magnification was 100X. Among the comet parameters, we report the % of DNA in the tail [tail DNA (%)]. This measures the extent of DNA damage induced by the chemical being tested. Images of 150 (50 cells/replica × 3 replicates) cells per concentration were analyzed for human lymphocytes. The median values of each concentration with respect to the comet parameter were calculated.

#### Measurement of DCFHDA oxidation by flow cytometry

Following treatment with PR-GO nanoparticles (0, 50, 100 and 250 μg/ml) for 3 h at 37°C, cells were washed twice with PBS and re-suspended in 500 μl (2 × 10^6^ cells) of PBS containing 25 μM DCFHDA. A set of three replicates per concentration were maintained for the experiment. After 15 minutes incubation at room temperature in the dark, the DCF fluorescence in cells was measured by flow cytometry (FACS Aria III flow cytometer, BD Biosciences, USA; excitation λ = 488 nm, emission λ = 530–540 nm) and 20,000 events were acquired for analysis. Results were expressed as the mean fluorescence intensity. The interference of nanoparticles in the assays was eliminated by maintaining sample blanks for all the concentrations tested. H_2_O_2_ (25 μM, 10 min in dark) treated cells were used as positive control for the experiment.

This study was carried out in strict accordance with the recommendations and guidelines of the Bioethics Committee for Animal and Human Research Studies, University of Calcutta (Sanction No. BEHR/AM/180609). Fresh blood samples for lymphocyte culture were obtained from healthy volunteers after obtaining written informed consent. Subsequent experiments were performed in vitro and did not involve any form of human intervention or studies in animal model and hence no additional clearance was required. The Bioethics Committee for Animal and Human Research Studies, University of Calcutta was fully aware of and sanctioned this present research work.

## Results and discussion

### Physico-chemical characterization of crude polysaccharide

**Fig 1:**
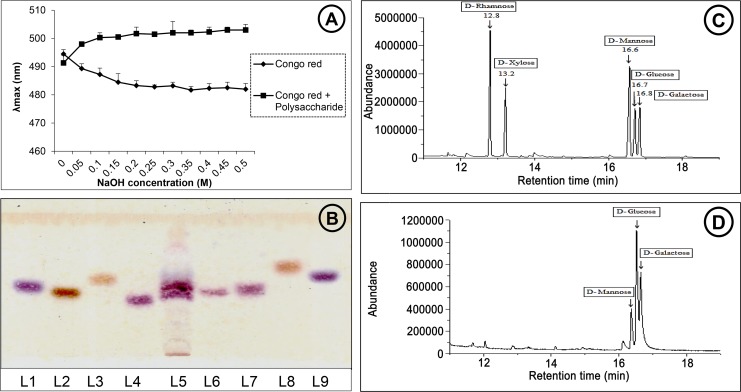
A: Changes in the absorption maximum of the congo red-polysaccharide complex at various concentrations of sodium hydroxide solution; B: HPTLC analysis for monosaccharide composition of the polysaccharide. L1: L-Arabinose L2: D-Fructose, L3: D-Fucose, L4: D-Galactose, L5: Polysacchaide, L6: D-Glucose, L7: D-Mannose, L8: D-Rhamnose, L9: D-Xylose; C: GC-MS of five standard monosaccharides and D: GC-MS of polysaccharide from *P*. *flabellatus*.

the crude poloysaccharide that nwas isolated from the dried basidiocarps of *P*. *flabellatus*, gave a yield of 4.5 ± 0.51% dry weight. it consisted mainly of carbohydrate (43.75 ± 3.25%) with low amount of protein (12.5%). No phenols were detected in the polysaccharide. Total glucan content of the polysaccharide was 17.68 ± 1.89% among which 1.29 ± 0.31% were α-glucans and 16.39 ± 2.2% were β-glucans. To further investigate the physical structure of polysaccharide, Congo red assay was performed which showed a bathocromic shift from 491 nm to 500 nm (Fig 1A)indicating the presence of triple helix of β-1, 3–1, 6-glucans as Congo red solely interacts with triple-helical polysaccharides [54]. HPTLC (Fig 1B) and GC-MS (Fig 1D) analysis indicated that the fraction was heteroglucan. Mannose, glucose and galactose residues were detected in the ratio of 18: 49.3: 32.7 from GC-MS analysis data.

### Production and characterization of PR-GO

#### Production of PR-GO nanosheets

**Fig 2:**
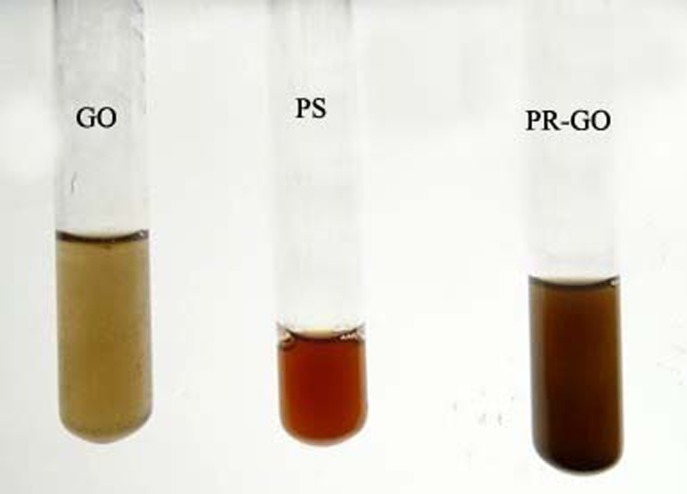
From left, dispersed and exfoliated graphene oxide, aqueous solution of the polysaccharide, polysaccharide-reduced graphene oxide.

The formation of PR-GO nanoparticles was visually observed by monitoring three flasks, containing graphene oxide solution (GO), polysaccharide solution (PS) and the reaction mixture of the polysaccharide with graphene oxide respectively. Only the reaction mixture displayed a time dependent colour-change, while the polysaccharide and the graphene oxide solutions retained their original colours (Fig 2). the initial reddish brown mixture became blackish brown, then a stable black with time at room temperature. The appearance of the dark brown colour indicated the occurrence of the reaction and the formation of PR-GO nanosheets [55,56].

#### Particle size measurement of the PR-GO by dynamic light scattering and observation of nanosheets by TEM

**Fig 3:**
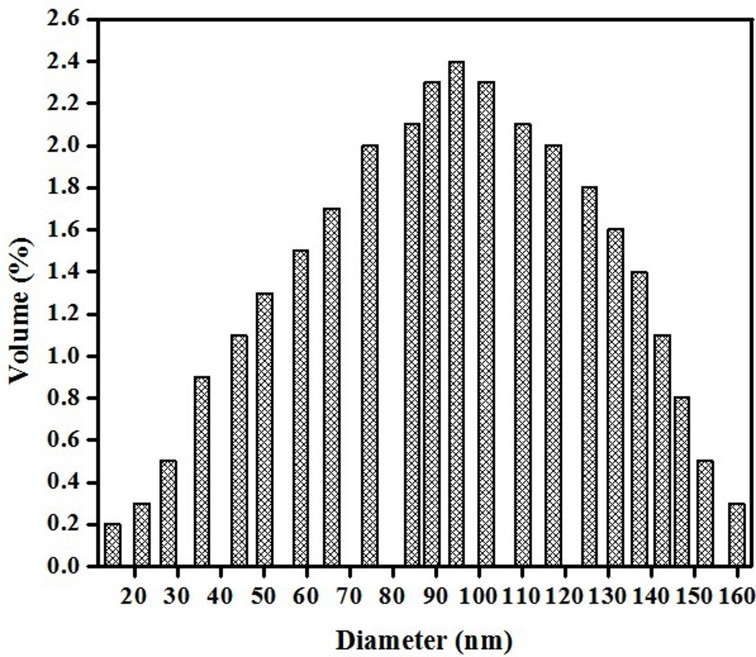
DLS elucidation of the synthesized PR-GO.

Particle size of PR-GO was determined by dynamic light scattering measurement. The size of the particles were found to be in the range of 15 to 155 nm (Fig 3). The size of the synthesized nanoparticles were further confirmed by the transmission electron microscopic (TEM) analysis. The average diameter of these PR-GO nanoparticles was of 65 ± 5 nm.

**Fig 4:**
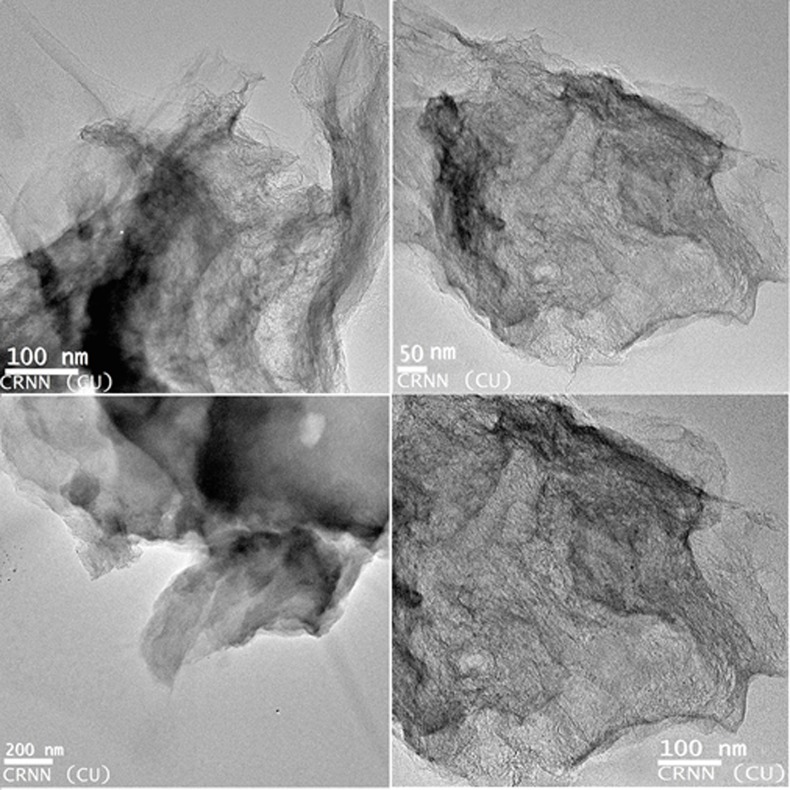
TEM image of PR-GO.

TEM image shown in Fig 4 captured PR-GO nanosheets of varied sizes, which arose from the bio-reduction of graphene oxide by polysaccharide at room temperature.

#### XRD analysis of the PR-GO

**Fig 5:**
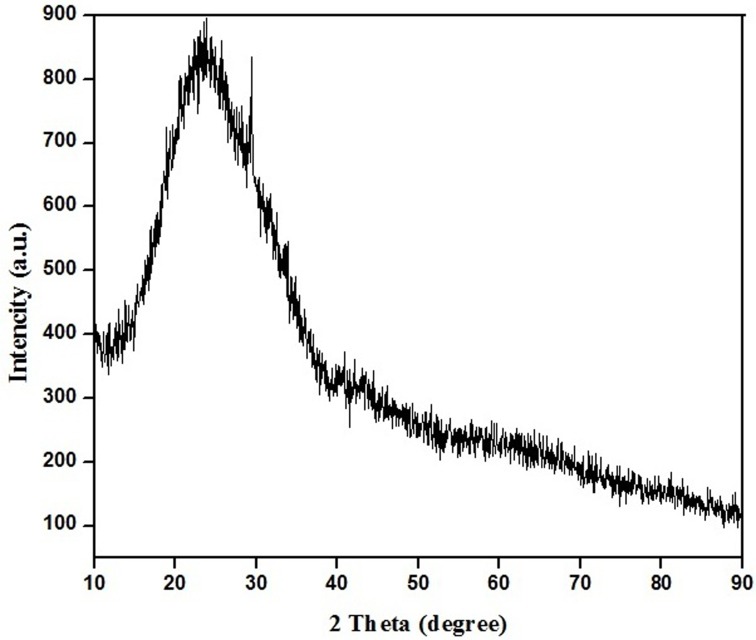
XRD spectrum of PR-GO nanosheets.

X-ray diffraction study was used to confirm the reduction of graphene oxide to crystalline graphene. XRD pattern that was obtained has been represented in the Fig 5. The XRD patterns clearly showed that the PR-GO nanosheets were formed by the bioreduction. The XRD pattern of pristine graphite exhibited a basal 002 reflection peak at 2θ = 26.6° (d-spacing = 0.335 nm). However, after oxidation of pristine graphite to GO, the 002 reflection peak shifted to the lower angle at 2θ = 11.32° (corresponding to a d spacing of 0.7816 nm), where the d-spacing increases due to the intercalation of oxygen functionalities in between the basal plane of graphite [57,58]. However, in our experiment, the appearance of a broad peak centered at 2θ = 23.5° indicated the presence of stacked graphene layers [59], validating the formation of few layer graphene [60].

#### EDX observation of PR-GO

**Fig 6:**
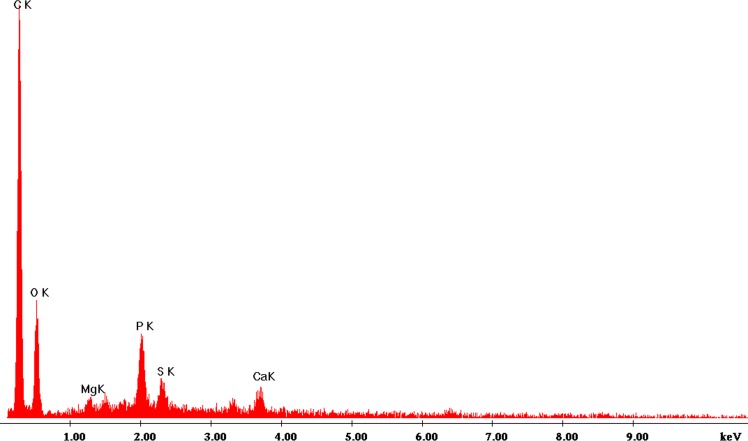
EDX spectrum of PR-GO nanosheets.

The Energy-dispersive X-ray (EDX) spectrum (Fig 6) recorded in spot-profile mode showed strong signals from the carbon atoms in the nanosheets.Sharp optical absorption peaks of carbon and oxygen in the range of 0 to 1 keV signified the presence PR-GO nano-crystallites.

#### FTIR analysis of crude polysaccharide of *Pleurotus flabellatus*, GO and PR-GO

**Fig 7:**
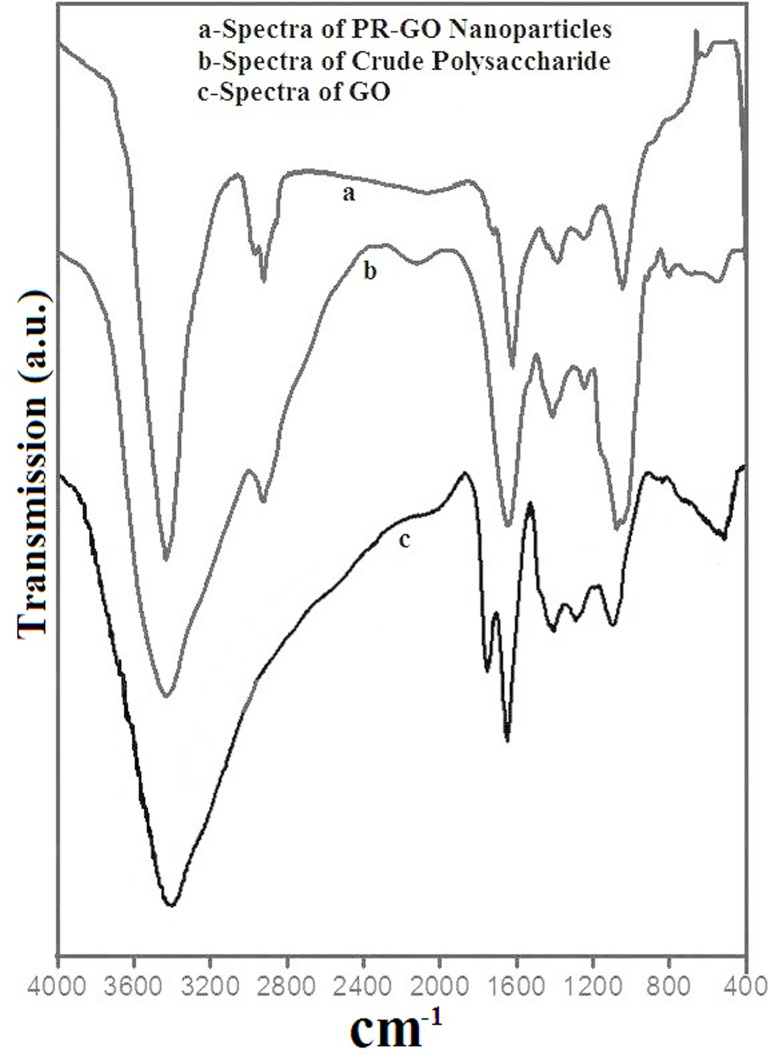
Fourier transform infrared spectra of (a) PR-GO; (b) Crude polysaccharide and (c) GO.

Fourier transform infrared spectroscopy deals with the stretching, bending, bonding and wiggle responses of molecular species in a given sample specimen. FTIR absorption spectra of the biosynthesized, vacuum dried GO, PR-GO nanosheets and crude polysaccharide of *Pleurotus flabellatus* have been shown in Fig 7. Each spectra showed an intense band at around ~3430 cm^-1^ due to the presence of O-H stretching [61]. The presence of a new peaks at around 2920 cm^-1^ were owing to the absorption of the polysaccharide extract containing phenolic compounds on the surface of graphene. So, from the above observation it is clear that the increased reduction time leads to a greater removal of the oxygen functionalities from the GO surface, indicating a successful reduction of GO to PRGO [62]. However, the peak at 1625 cm^-1^ attributed to the aromatic C = C groups present in all three spectra. This suggests that the frame of sp^2^ carbon atoms after reduction by crude polysaccharides was retained well, as before [63]. The characteristic peaks for C = O stretching vibration appeared at 1746 cm^-1^ in case of GO, while they were completely absent in the spectra of PR-GO as well as crude polysaccharides [63]. The FTIR peaks at around 1250 cm^-1^ and 1060 cm^-1^, found in the spectra of GO and PR-GO nanoparticles, signified the epoxy C-O and alkoxy C-O stretching vibration, respectively [64]. However, the peak positions and intensities observed for GO changed significantly in case of PR-GO. This can also be attributed to the partial reduction of GO to graphene [56,60]. Bands observed at around 1420 cm^-1^ was due to N-H deformation [65] found in the spectra of crude polysaccharide, which shifted significantly to 1395.8 cm^-1^ (PR-GO spectra) due to the symmetric stretching of the carboxyl side groups of the polysaccharide molecules [66]. This indicated a strong interaction between the crude polysaccharide and the PR-GO nanoparticles. The spectral band around 1077 cm^-1^ of the polysaccharide spectrum indicated C-C and C-O stretching vibrations in pyranoid rings indicating pyranose form of sugar [67,68]. The bands visible between 500 and 749 cm^-1^ signified the presence of R–CH groups [69]. In the crude polysaccharide, a weak band at around 800 cm^-1^ signifies the presence of β-glycosidic bond and therefore indicated the existence of β-glucan. These analyses further corroborated our inference that the polysaccharide consists mainly of β-structures of pyranose sugars and that the interaction of GO with the crude polysaccharide formed PR-GO nanosheets.

#### Raman spectra of PR-GO nanosheets

**Fig 8:**
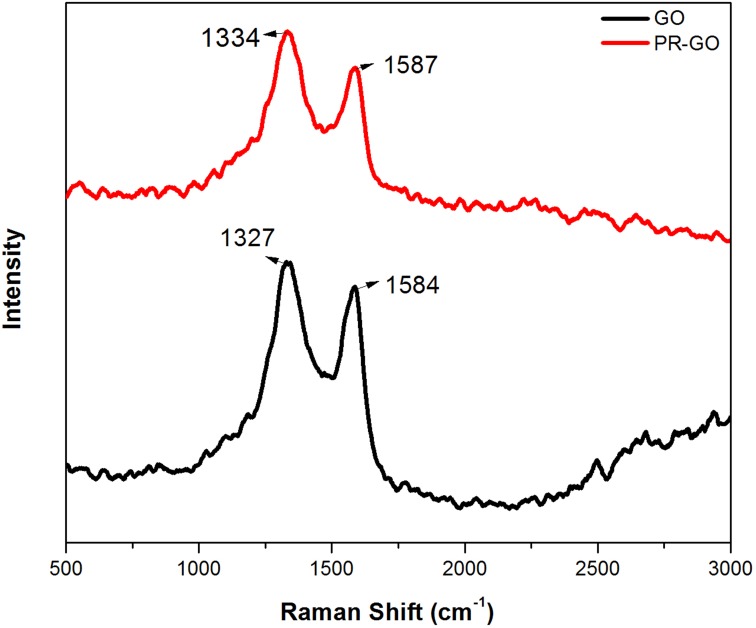
Raman Spectrum of PRGO (Curve A) and GO (Curve B).

Raman scattering from a material strongly depends on its electronic structure. Hence, it is very useful for characterization of materials and has been extensively used to investigate the structures of graphite and graphene like materials. In particular, Raman spectroscopy has been used to study the extent of structural changes undergone during reduction of GO. The relative intensities of the two fundamental vibrational bands between 1100 and 1700 cm^-1^ in the Raman scattered light observed for both GO and PR-GO are crucial for this purpose (Fig 8). The band with the lower Raman shift is known as the D-band and the other one near 1600 cm^-1^ is the G-band. The D-band originates from a breathing mode vibration involving κ-point mode phonons of A_1g_ symmetry, while the G-band arises from first order scattering by vibrational modes of the sp2 carbons of GO and PR-GO with E_2g_ symmetry. Thus, the D-band arises from vibration of sp2 carbon clusters, and hence the relative increase of the intensity of the D-band compared to the G-band in a sample would indicate the presence of greater portions of such sp2 bonded carbon clusters without defects. In the context of comparison of the Raman spectra of GO and PR-GO, the increase of purely graphene like domains in the PR-GO was expected to cause an increase of the ratio of the D and G-band intensities because of the removal of oxygen containing moieties from GO. This has actually been observed in our samples. The accompanying figure shows the fundamental Raman bands in both the GO and the PR-GO, shifted vertically by an arbitrary amount for clarity. The D-band for GO occurs at 1327 cm^-1^, and in case of PR-GO it shows a shift to 1334 cm^-1^. For the G band the positions of the vibrational band peaks are 1584 cm^-1^ for GO and 1587 cm^-1^ for PR-GO. Also, the D-band becomes relatively more prominent in the Raman spectrum of PR-GO. The ratio of the peak intensities of the two bands increases from 1 in GO to about 1.15 in PR-GO. The lower ratio for GO indicates the destruction of the sp2 orbitals due to the linkage of the C atoms with oxidizing groups. Our observation was also consistent with those obtained by other workers using various other green reduction techniques [56,70]. In the work of Xing-Hua Xia et. al., [71] it was shown that the 2D peak becomes weak and broadened as we go from graphite to reduced graphene oxide. In our work, also this line became so weak and diffused that no 2D peak at 2750 cm^-1^ region could be observed.

### Bio-compatibility of PR-GO nanosheets

While carbon based nanomaterials, including graphene oxide are potential candidates for biomedical applications, their bio-compatibility is of prime concern, as several studies have reported the general toxicity of graphene based nanomaterials [28]. Hence, in addition to synthesis of PR-GO nanosheets, considerable emphasis has been given to safety assessment of these particles. A battery of tests has been used to determine the toxicity of the synthesized particles in human PBMCs.

#### Cytotoxic effect of PR-GO nanosheets on human PBMCs

**Fig 9:**
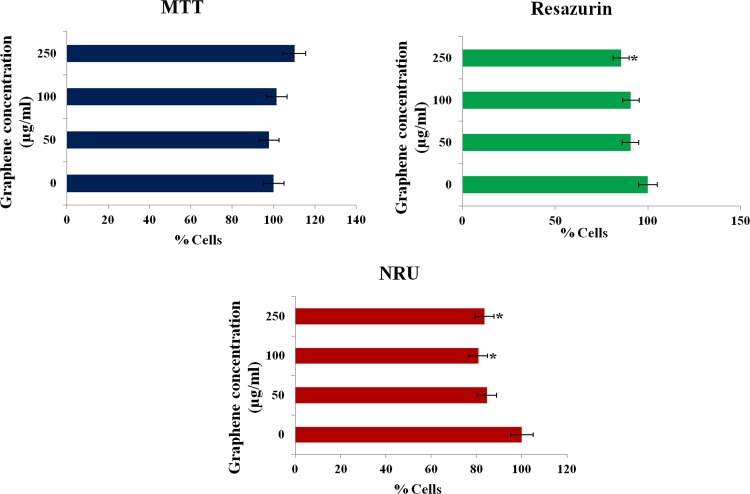
Cytotoxicity of PR-GO in terms of MTT, Resazurin and neutral red uptakeassays.

**Fig 10:**
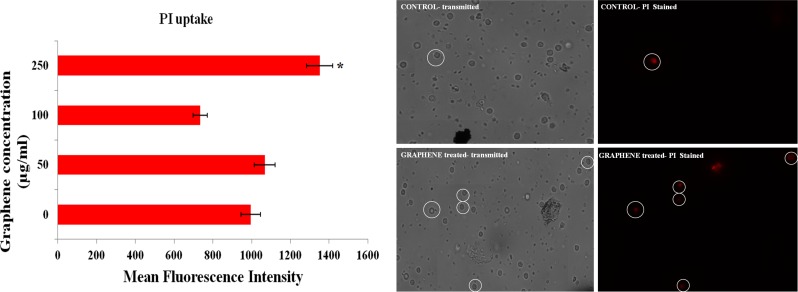
Flow cytometry and confocal microscopy of human PBMC treated with different concentrations of PR-GO.

**Fig 11:**
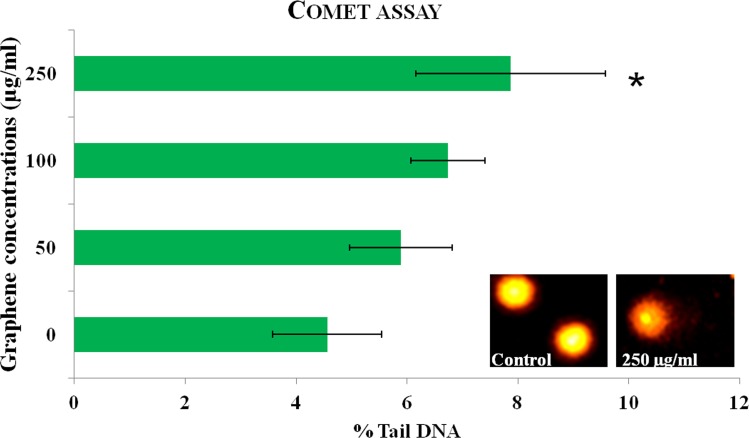
Genotoxic effects of different concentrations of PR-GO on human PBMCs.

The cytotoxic potential of PR-GO nanosheets in human PBMCs was evaluated using more than one endpoint, to avoid false positive/ negative results [72]. MTT assay was used to study alteration in mitochondrial activity [73] in cells exposed to PR-GO nanosheets. From the results of MTT assay, no significant change in mitochondrial activity could be observed (Fig 9). The results of neutral red uptake assay indicated loss of lysosomal integrity, induced by PR-GO nanosheets at concentrations of 100 μg/ml and above (Fig 9). [74,75]. The effect of PR-GO on cell membrane integrity was studied using flow cytometry and confocal microscopy (Fig 10). PI uptake increased significantly at the highest concentration tested (250 μg/ml), indicating loss of membrane integrity. In resazurin assay, magnitude of dye reduction gives an overview of the cell’s metabolic condition. In the present study resazurin assay revealed significant alteration in metabolic activity at the highest treatment concentration (250 μg/ml) as compared to control (Fig 11). Considering at least one of the tests indicated toxic response, with respect to membrane integrity, mitochondrial function, lysosome integrity, and metabolic activity; PR-GO could be considered safe below concentrations of 100 μg/ml.

#### DNA damage induced by PR-GO in human PBMCs

In addition to cytotoxicity, genotoxicity of the particles was evaluated using comet assay. Genotoxicity assessment of particle could provide valuable information regarding DNA damage that might lead to better understanding of mutation and cancer. Genotoxicity of various graphene oxides has been reported by some authors [76–78]. In the present study, DNA fragmentation induced by PR-GO in PBMCs was statistically significant (*P* ≤ 0.05) at treatment dose 250 μg/ml. Treatment doses 50 and 100 μg/ml induced DNA damage, but was not statistically significant when compared to control (Fig 11). Significant DNA damage was induced by H_2_O_2_ (% Tail DNA: 55±8) and was used as a positive control for the experiment.

#### Generation of ROS in human PBMCs

**Fig 12:**
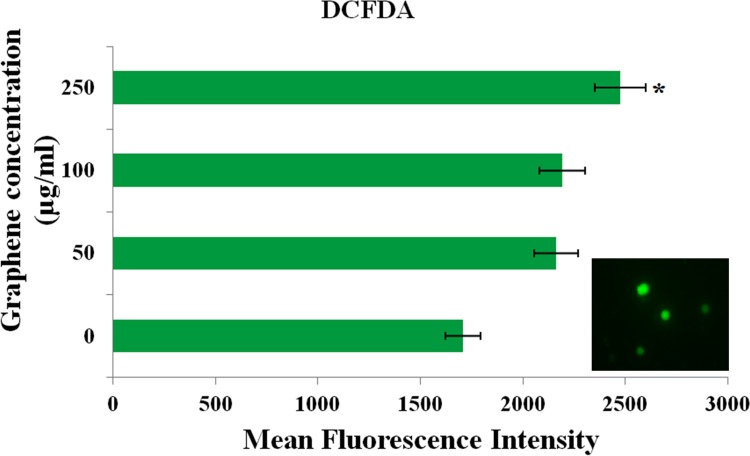
ROS generation in human PBMCs induced by PR-GO.

In addition to direct DNA damage, certain nanoparticles are known to induce oxidative stress. Flow cytometric analysis indicated concentration dependent increase in ROS production (Fig 12). There was a ^˷^1.8-fold increase in ROS generation at the highest concentration tested (250 μg/ml), and ^˷^6-fold increase in the positive control (H_2_O_2_). In a study by Chang et al. (2011), the authors suggested of a dose-dependent oxidative stress in A549 cells, despite little/absence of cytotoxic response. In another study [21] the authors revealed of GO internalization, cell-cycle alterations, apoptosis and oxidative stress.

## Conclusion

In conclusion, a green approach to the synthesis of graphene nanosheets is reported using exfoliated graphene oxide as the precursor and a crude fungal polysaccharide as the reducer. The use of an environment-friendly reducing and capping agent is what this method derives its merit from. The method should find practical applications in bulk-synthesis of graphene nanosheets. Also, from all the toxicity endpoints studied in human PBMCs, this newly synthesized nanoparticle could be considered biologically safe at concentrations below 100 μg/ml.
